# Complete Structural Elucidation of Monophosphorylated Lipid A by CID Fragmentation of Protonated Molecule and Singly Charged Sodiated Adducts

**DOI:** 10.1021/jasms.2c00269

**Published:** 2022-12-20

**Authors:** Ibrahim Aissa, Ágnes Dörnyei, Viktor Sándor, Anikó Kilár

**Affiliations:** † Department of Analytical and Environmental Chemistry, Faculty of Sciences, University of Pécs, Ifjúság útja 6, H-7624 Pécs, Hungary; ‡ Institute of Bioanalysis, Medical School and Szentágothai Research Centre, University of Pécs, Szigeti út 12, H-7624 Pécs, Hungary

## Abstract

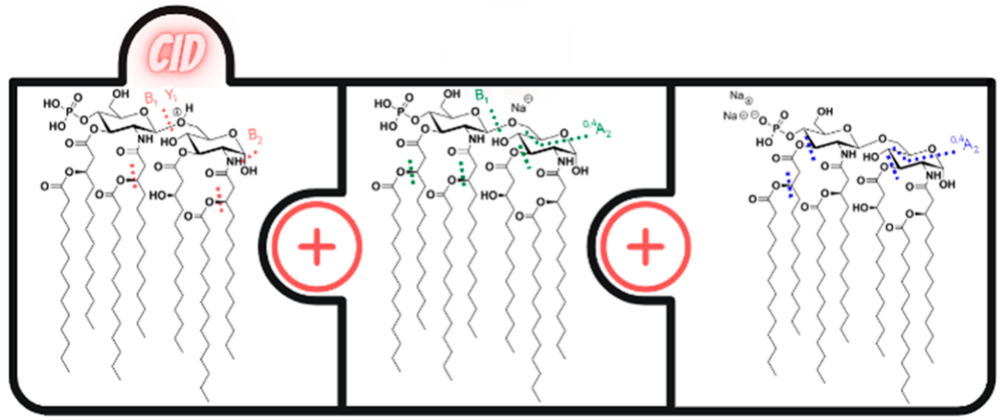

Lipid A, the inflammatory portion of lipopolysaccharides (LPS, endotoxins), is the main component of the outer membrane of Gram-negative bacteria. Its bioactivity in humans and animals is strictly related to its chemical structure. In the present work, the fragmentation patterns of the singly charged monosodium [M + Na]^+^ and disodium [M – H + 2Na]^+^ adducts, as well as the protonated form of monophosphorylated lipid A species were investigated in detail using positive-ion electrospray ionization-based tandem (MS/MS) and multistage mass spectrometry (MS^n^) with low-energy collision-induced dissociation (CID). Several synthetic and native lipid A samples were included in the study. We found that the fragmentation pattern of disodiated lipid A is quite similar to that of the well-characterized deprotonated lipid A molecule (typically detected in the negative-ion mode), while the fragmentation pattern of monosodiated lipid A contains fragment ions similar to those of both protonated and deprotonated lipid A molecules. In summary, we propose a new mass spectrometry approach based on the fragmentation regularities of only positively charged precursor ions to dissect the location of the phosphate group and fatty acid moieties on monophosphorylated lipid A. Moreover, this study provides a better understanding of the so-called “chimera mass spectra”, which are commonly detected during the fragmentation of native lipid A samples containing both C-1 and C-4′ phosphate positional isomers but rarely identified in negative-ion mode.

## Introduction

Lipopolysaccharides (LPS), also known as endotoxins, are organic components of the outer membrane of Gram-negative bacteria. They are embedded in the outer layer of the membrane with their lipid A moiety, to which a polysaccharide chain is attached extending outward from the cell surface.^1^ Besides having essential functions for the bacterium, they play a key role in various bacterial infections, sepsis, and the development of septic shock.^2,3^ At the same time, the biological activities of different bacterial LPS molecules depend on fine structural requirements of both lipid A and polysaccharide parts.

The lipid A portion is responsible for the endotoxic activity of LPS.^4^ Over the decades, more and more lipid A variants have been successfully extracted from a variety of bacterial strains to perform structure elucidation and bioactivity profiling. One main reason for this is that understanding the structure and function of the lipid A moiety is a key strategic point in the development of new and innovative drugs, such as vaccine adjuvants^5,6^ or anticancer agents.^7^ In particular, monophosphoryl lipid A (MPLA), a detoxified analogue of lipid A manufactured from *Salmonella minnesota*, and other synthetic MPLA analogs, such as PHAD, 3D-PHAD, and 3D-(6-acyl)-PHAD manufactured based on the current good manufacturing practice (cGMP) guidelines, are used as adjuvants in many commercialized human vaccines.^8,9^

Structurally, lipid A is made up of a β-(1 → 6)-linked glucosamine disaccharide backbone phosphorylated at position C-1 and/or C-4′ and acylated at positions C-2, C-3, C-2′, and C-3′ of the saccharides.^10^ Naturally, lipid A is a mixture of tri-, tetra-, penta-, hexa-, and sometimes even hepta-acylated forms, meaning that three, four, five, six, or seven acyl chains (of variable length) are esterified to the disaccharide backbone. Primary acyl chains, which are directly esterified to the sugar moiety, are mostly hydroxylated, while the so-called secondary acyl chains form ester bonds with the hydroxyl groups of primary acyl chains.

Analysis of lipid A relies primarily on MS/MS and/or multiple-stage MS^n^ strategies to generate fragmentation profiles and multiple-level fragments for structural determination.^11,12^ Mostly, the negative-ionization mode is used because phosphate group(s) in lipid A can be easily deprotonated and the resulting anions [M – H]^−^ and/or [M – 2H]^2–^ show high detection sensitivity in MALDI or ESI MS. Specifically, negative-ion mode ESI MS/MS analysis is useful to gain information on the acylation profile of differently phosphorylated^13−15^ and even nonphosphorylated^16^ lipid A species in deprotonated form. However, in a native sample, the simultaneous presence of phosphate positional isomers (i.e., lipid A compounds containing the phosphate group at either C-1 or C-4′) results in a chimera mass spectrum (i.e., a mixture of mass spectra stemming from cofragmenting isobaric lipid A precursor ions), which is highly difficult to identify in the negative-ion mode due to overlapping mass spectra. On the other hand, the phosphorylation site (and partly the fatty acid composition) can be resolved from positive-ion mode fragmentation analysis of the protonated form^17^ [M + H]^+^ or the triethylammonium adduct^18,19^ [M + H + Et_3_N]^+^ of monophosphorylated lipid A species. During their CID fragmentation, an oxonium ion is formed, assigned as the distal sugar fragment; thus, the presence or absence of the phosphate group connected to it (at position C-4′) can be determined. However, the reason that researchers usually do not perform MS/MS analysis of these two types of precursor ions in the positive-ionization mode is the poor intensity ratio of the [M + H]^+^ ion (compared to that of the [M – H]^−^ in the negative mode), as well as the strong memory effect in mass spectrometers resulting from the adduct forming triethylamine.^20^ Nevertheless, there are several studies that report on the tandem MS analysis (with PD, FAB, MALDI, or ESI technologies) of the monosodium adduct^21−27^ [M + Na]^+^ of phosphorylated and nonphosphorylated lipid A species because this type of precursor ion is usually recovered in higher abundance (especially for nonphosphorylated species). In those studies, the formation of an oxonium ion was observed as well, which was usually used only to confirm the interpretation of the negative-ion mass spectra for the acylation pattern determination of lipid A species. In some cases, disodiated lipid A adducts, such as [M – H + 2Na]^+^ or [M – H – PO_3_H + 2Na]^+^, were also detected in positive-ion experiments of mono- and bisphosphorylated lipid A species,^21,23−25,28^ albeit those type of ions had not been fragmented.

Here, we focus on the positive-ion mode ESI MS/MS and MS^n^ analysis of a set of synthetic and naturally sourced (from *E. coli* O83) monophosporylated lipid A compounds recovered as mono- and disodium adducts, as well as a protonated molecule. CID patterns of these three precursor ion types detected in a single ionization mode were systematically investigated to gain insight into the fragmentation behavior of such ions and summarize cleavage rules that can be applied for the structural analysis of complex lipid A samples containing a mixture of 1- and 4′-monophosphorylated isobaric species.

## Experimental Section

### Chemicals and Samples

Methanol (MeOH) and dichloromethane (DCM) (LC–MS Chromasolv grade) were purchased from Sigma-Aldrich (Steinheim, Germany), and ammonium formate (LC–MS Chromasolv grade) was from Fluka (Seelze, Germany).

### Synthetic Lipid A Standards

Four types of synthetic monophosphoryl lipid A standards, such as monophosphoryl 3-deacyl penta-acyl lipid A (3D-PHAD, Pat No. 9,241,988), monophosphoryl 3-deacyl hexa-acyl lipid A (3D(6-acyl)-PHAD), monophosphoryl lipid A (PHAD), and monophosphoryl lipid A-504 (PHAD-504), were purchased from Sigma-Aldrich, the exclusive Avanti Polar Lipids provider (Alabaster, AL, USA) in Hungary. About 0.1 mg of each standard was dissolved in 1 mL of MeOH:DCM (70:30, v/v) mixture. Then, 5 mg of ammonium formate was added, and samples were vortexed and subsequently put in an ultrasonic bath (5 min) to be highly homogeneous. Next, 300 μL of each sample was introduced into sealed glass vials, and 700 μL of methanol was added. After vortexing, the samples were ready for injection.

### Lipid A Isolation from *E. coli*

The bacterial strain of *Escherichia coli O83* (*E. coli* O83) was cultured at 37 °C in a laboratory fermentor on Mueller–Hinton broth at pH 7.2 until it reached the late logarithmic phase (about 10 h) and then collected by centrifugation. LPS was extracted from acetone-dried organisms by the classical hot phenol/water procedure^29^ in a yield of 5% of bacterial cell dry mass and was lyophilized. Lipid A was released from LPS by mild acid hydrolysis with 1% (v/v) AcOH (pH 3.9) at 100 °C for 1 h; then, the solution was centrifuged (8000*g*, 4 °C, 20 min). The sediment containing lipid A was washed four times with distilled water and lyophilized. About 0.1 mg of lipid A was dissolved in 1 mL of MeOH:DCM (70:30, v/v) mixture. Then, 5 mg of ammonium formate was added, and the sample was vortexed and subsequently put in an ultrasonic bath (5 min). Next, 300 μL of the sample was introduced into a sealed glass vial, and 700 μL of methanol was added. A small amount of NaCl (about 0.5 mg) was added to promote sodium cation attachment to the phosphorylated lipid A molecules. After vortexing, the sample was ready for injection.

### Mass Spectrometry Analysis

Accurate Mass Q-TOF measurements were performed in the positive-ion mode using a 6530 Accurate-Mass Quadrupole Time-of-Flight (Q-TOF) (Agilent Technologies, Singapore). The injection of samples was carried out with a UHPLC autosampler (Agilent Technologies, Waldbronn, Germany) with a volume of 1 μL. Positive-ion mass spectra were recorded in the mass range of *m*/*z* 50–2200 at a measuring frequency of 1000 transients/s and a detection frequency of 4 GHz. The Agilent Jet Stream ion source was set up using the following conditions: the pressure of nebulizing gas (N_2_) was 30 psi, the temperature of drying gas (N_2_) was 300 °C with a flow rate of 7 L/min, and the temperature of the sheath gas was 300 °C with a flow rate of 11 L/min. The capillary voltage was configured to 3500 V, the nozzle voltage to 2000 V, the fragmentor potential to 100 V, and the skimmer potential to 70 V. For the targeted MS/MS analysis, the following parameters were used: mass range, *m*/*z* 50–2000; acquisition rate, 333.3 ms/scan; quadrupole isolation width, narrow (*m*/*z* 1.3); collision energies, 30–100 eV.

ESI ion trap MS^n^ analysis was performed in the positive-ion mode using an MSD Trap XCT Plus mass spectrometer (Agilent Technologies, Germany), controlled with the Agilent LC/MSD Trap Software 5.3. A syringe pump set at a flow rate of 3 μL/min was used for direct infusion of the samples into the ion source. The electrospray capillary high voltage was applied at 3500 V. Nitrogen was used both as a nebulizer (15 psi, 5 L/min) and dry gas (at 325 °C). Spectra were scanned in the range *m*/*z* 50–2200. The mass isolation window for precursor ion selection was set to 4 Da in the multiple-stage analysis. Each precursor ion was excited by resonant excitation voltage fixed between 0.5 and 1.5 V, according to the intensity of the parent ion. Positive-ion mass spectra, including collision-induced dissociation (CID) spectra, were averaged over 5–20 scans, depending on the relative abundances of the precursor ions.

### Data Evaluation

The evaluation of the MS/MS and MS^n^ mass spectra was made by considering the monoisotopic masses of the fragment ions and the neutral losses based on the known composition and structure of the precursor ions. The following abbreviations were used to indicate the chemical compositions during the interpretation of mass spectra: GlcN (2-amino-2-deoxy-glucopyranose), C12:0 (dodecanoic acid or lauric acid), C14:0(3-OH) (3-hydroxytetradecanoic acid or hydroxymyristic acid), C14:0 (tetradecanoic acid or myristic acid), and C14:1 (tetradecenoic acid or unsaturated myristic acid). The classical nomenclature for glycoconjugates and cross-ring fragmentation nomenclature described by Domon and Costello^30^ was adopted to designate the fragment ions.

## Results and Discussion

The ESI-Q-TOF mass spectrum of each lipid A sample recorded in the positive-ion mode presented three ions corresponding to the protonated lipid A molecule [M + H]^+^, the monosodium adduct [M + Na]^+^, and the disodium adduct [M – H + 2Na]^+^ (Figure S1). Among them, the [M + Na]^+^ ion was dominant, while the [M – H + 2Na]^+^ ion was the least abundant. It should be noted here that the formation of the [M + H]^+^ ion resulted from the in-source decomposition of the ammonium adduct [M + NH_4_]^+^ of lipid A. A direct comparison of the ionization sensitivity of the same lipid A in the positive mode versus the conventional negative mode (Figure S2) showed that the ionization efficiency (i.e., the amount of ions generated from a specific compound in the ionization source) of both modes was in the same order of magnitude; however, due to the multiple ion formation in the positive mode, ion abundancies were about 1 to 2 orders of magnitude smaller than the negative mode.

Next, the above-mentioned three positively charged ions were selected as precursors, and their fragmentation patterns obtained by low-energy CID were investigated. To the best of our knowledge, a systematic MS/MS study of sodiated adducts and the protonated molecule of lipid A has not yet been performed, and their usefulness for the full structure elucidation of monophosphorylated lipid A species has not yet been investigated in comparison to the traditional negative mode studies.

### CID Fragmentation Pattern of the Protonated Monophosphorylated Lipid A Molecule

**Figure 1:**
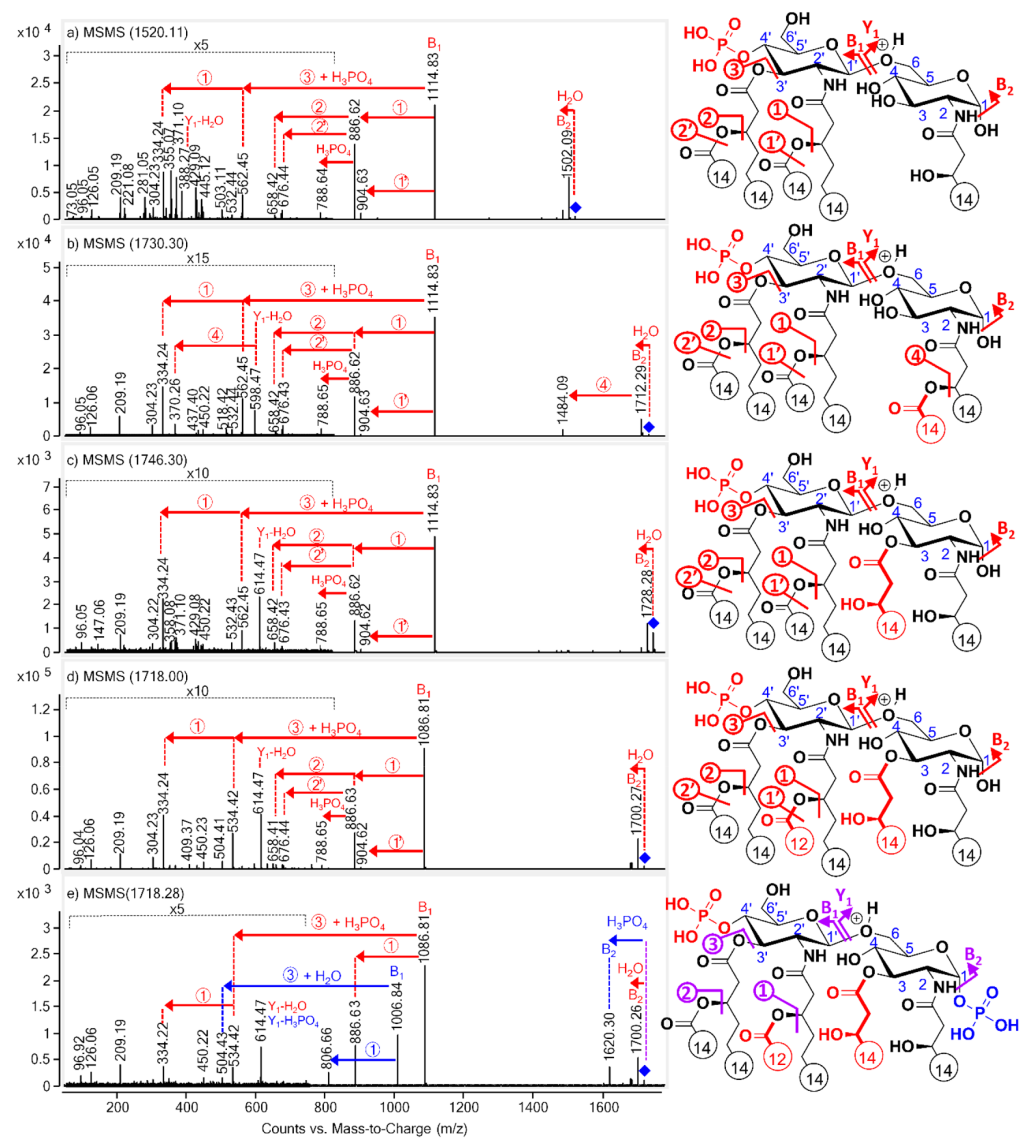
ESI-Q-TOF MS/MS mass spectra of the [M + H]^+^ lipid A precursor ions of (a) 3D-PHAD (*m*/*z* 1520), (b) 3D(6-acyl)-PHAD (*m*/*z* 1730), (c) PHAD (*m*/*z* 1746), (d) PHAD-504 (*m*/*z* 1718), and (e) *E. coli* O83 (*m*/*z* 1718) with the indication of cleavage sites in the structures. Since both 1- and 4′-monophosphoryl species were present in the *E. coli* sample, its structure has been sketched carrying two phosphate groups for information purposes only. Red signs match with the 4′-monophosphoryl species, blue signs with the 1-monophosphoryl species, and purple signs refer to both isomeric species. Fatty acyl chain lengths are given by numbers, and colored structural parts indicate the differences between the lipid A congeners.

Figure 1 shows the ESI-Q-TOF MS/MS mass spectra of the [M + H]^+^ precursor ions of the four lipid A standards and hexa-acylated *E. coli* O83 lipid A. Typically, the most intense fragment ion (at *m*/*z* 1115, except for PHAD-504 and *E. coli*, for which it was at *m*/*z* 1087) resulted from the cleavage of the glycosidic bond connecting the two glucosamine units, also known as the B_1_ ion (inter-ring cleavage). However, it is important to note that for the native sample, being a mixture of 1- and 4′-monophosphoryl species, two B_1_ ions—a phosphorylated one (*m*/*z* 1087) and a nonphosphorylated one (*m*/*z* 1007)—appeared, although the intensity of the latter was smaller. From the B_1_ ion, loss of the secondary fatty acid at C-2′ occurred for all compounds, resulting in the moderate-intensity fragment ion at *m*/*z* 887, except for the 1-monophosphoryl lipid A species present in the *E. coli* sample (Figure 1e), for which it gave an ion at *m*/*z* 807. The location of this fatty acid release, i.e., at the C-2′-branched position, could be easily identified by comparing the mass difference related to this elimination in the case of PHAD-504 (Δ*m* = 200 u, reflecting the loss of a C12:0) with that observed for the other three lipid A standards (Δ*m* = 228 u, corresponding to the elimination of a C14:0 from the same position). The C-2′ secondary fatty acid was also lost as a ketene resulting in the low-intensity ion at *m*/*z* 905. Furthermore, for all synthetic derivatives, low-intensity ions appeared at *m*/*z* 789, 677, and 659 formed by the loss of phosphoric acid (H_3_PO_4_, 98 u), the C-3′ secondary fatty acid as a ketene (210 u), and an acid (228 u), respectively, from the triacylated B_1_ ion (*m*/*z* 887). The fragment ion at *m*/*z* 562 (or at *m*/*z* 534 for PHAD-504 and *E. coli*) resulted via the cleavage of the C14:0(3-O-C14:0) at the C-3′ position (Δ*m* = 454 u) along with phosphoric acid elimination (98 u) from the B_1_ ion. Subsequently, the formation of the fragment ion at *m*/*z* 334 could be due to the further loss of C14:0 (or C12:0 for PHAD-504 and *E. coli*) at the C-2′ secondary position. Furthermore, each tandem mass spectrum also exhibited another inter-ring cleavage product with low intensity (i.e., appearing at *m*/*z* 388 in Figure 1a, 598 in Figure 1b, and 614 in Figure 1c–e) known as a Y-type ion. More precisely, it is an internal fragment ion since both glycosidic bonds were cleaved. However, we are going to refer to it hereinafter as the Y_1_* ion which had lost the water molecule (or any substituent attached) at C-1. In the case of 3D(6-acyl)-PHAD, fatty acid loss at the C-2 secondary position (C14:0, 228 u) from this Y-type ion was also detected by a small fragment at *m*/*z* 370 (Figure 1b). Apart from the above-mentioned ions, a high-intensity fragment due to water loss (18 u) from the C-1 position of the precursor ion appeared for all derivatives, whereas a phosphate group loss (98 u) from the same position (resulting in an ion at *m*/*z* 1620) could be observed only for the *E. coli* lipid A sample (Figure 1e). Such ions, resulting from the cleavage of the glycosidic bond of the reducing end sugar, are called B_2_ ions. Here again, as with the Y-type ion of 3D(6-acyl)-PHAD, the cleavage of the C14:0 from the C-2 secondary position could also be observed from the B_2_ ion by the low-intensity ion at *m*/*z* 1485 (Figure 1b).

Next, the fragmentation patterns of the intact B_1_ ions selected as precursor ions using ESI-IT MS^3^ measurements were investigated. In the MS^3^ mass spectra of the B_1_ ions of the standards (Figure S3) and of 4′-monophosphorylated lipid A from *E. coli* (Figure S4a), the base peak at *m*/*z* 887 resulted from the release of the C-2′ secondary fatty acid as an acid, next to which a low-intensity ion appeared at *m*/*z* 905 resulting from the loss of the same fatty acid as a ketene. Next, two ions (*m*/*z* 789 and 807) with low intensities were formed via the loss of phosphoric acid (H_3_PO_4_, 98 u) and metaphosphoric acid (HPO_3_, 80 u) from the nonintact B_1_ ion at *m*/*z* 887. Moreover, from the same (triacylated) B_1_ ion, the C-3′ secondary fatty acid was eliminated both as an acid (*m*/*z* 659) and a ketene (*m*/*z* 677). Further cleavage products were formed by the release of the C-3′ primary fatty acyl residue as an acid (*m*/*z* 433) and a ketene (*m*/*z* 451). Loss of the phosphate group from the di- and monoacylated B_1_ ions resulted in the small-intensity ions at *m*/*z* 561 and 335, respectively. The MS^3^ mass spectrum of the B_1_ ion at *m*/*z* 1007 of the 1-monophosphorylated lipid A from *E. coli* O83 (Figure S4b) was characterized by the same series of fatty acyl losses and intensity ratios as observed for the 4′-monophosphorylated species, except that elimination of the C14:1 residue as a ketene from the C-3′ position was not detected. Also, there was a lack of ions resulting from the loss of the phosphate group, which was obvious since the distal sugar unit (representing the precursor ion) was originally not phosphorylated at the C-4’ position.

### CID Fragmentation Pattern of the Monosodium Adduct of Monophosphorylated Lipid A

**Figure 2:**
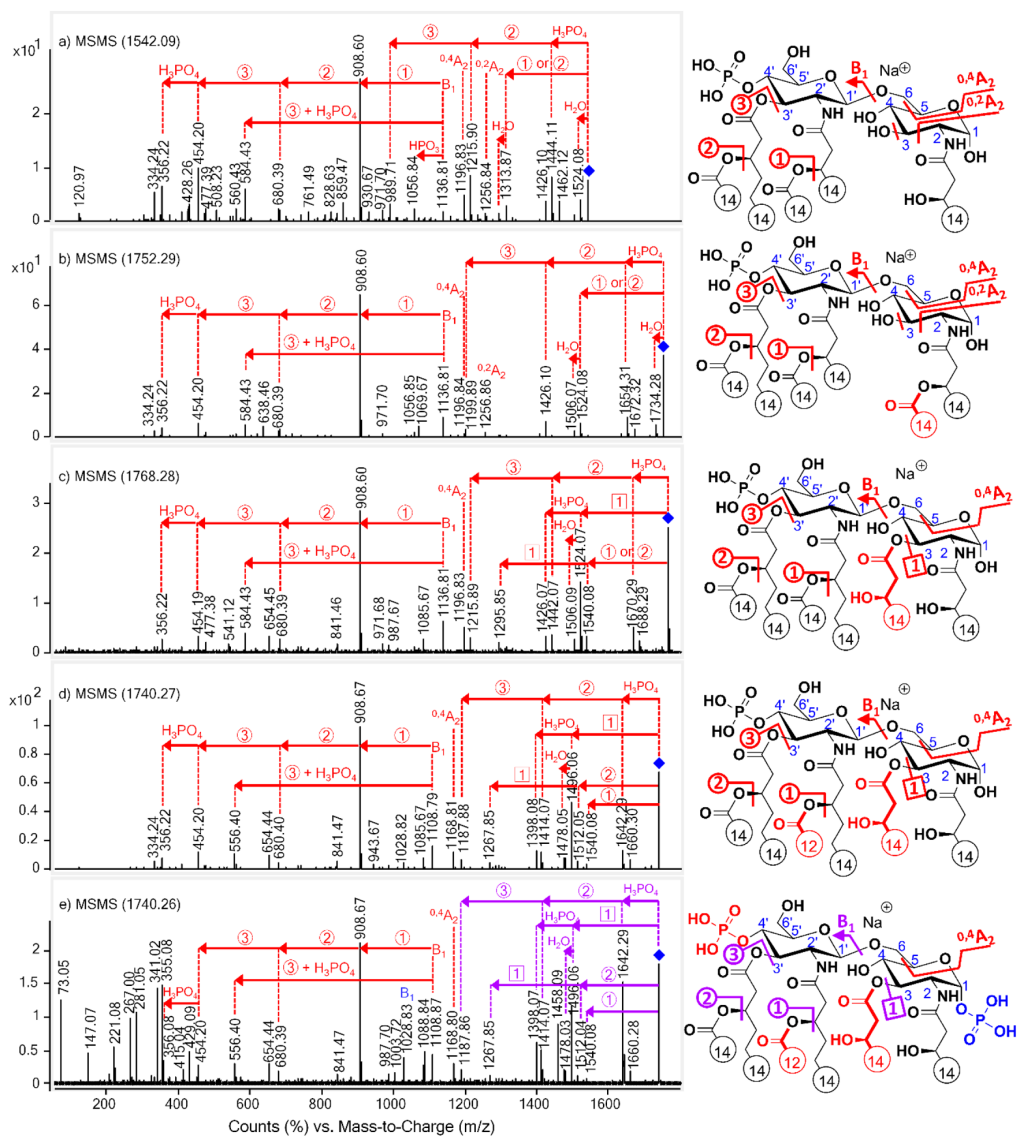
ESI-Q-TOF MS/MS mass spectra of [M + Na]^+^ lipid A precursor ions of (a) 3D-PHAD (*m*/*z* 1542), (b) 3D(6-acyl)-PHAD (*m*/*z* 1752), (c) PHAD (*m*/*z* 1768), (d) PHAD-504 (*m*/*z* 1740), and ESI ion trap MS/MS mass spectrum of (e) *E. coli* O83 (*m*/*z* 1740), with the indication of cleavage sites by numbers in circles or squares in the structures. Since both 1- and 4′-monophosphoryl species were present in the *E. coli* sample, its structure has been sketched carrying two phosphate groups for information purposes only. Red signs match with 4′-monophosphoryl species, blue signs match with 1-monophosphoryl species, and purple signs refer to both isomeric species. Fatty acyl chain lengths are given by numbers, and colored structural parts indicate the differences between the lipid A congeners.

The ESI-Q-TOF MS/MS mass spectra of the [M + Na]^+^ precursor ions are displayed in Figure 2. In each MS/MS mass spectrum, several first-generation ions appeared resulting from the competitive elimination of the phosphate group, the secondary fatty acids at the C-3′ or C-2′ positions and a water molecule (for the 3-deacyl species) or a C14:0(3-OH) (for the 3-acyl compounds) at C-3 from the precursor ion. Furthermore, second- and third-generation fragments could also be identified.

Namely, the phosphoric acid loss was complemented with the serial release of the C-3′ secondary and primary fatty acids. The loss of the secondary fatty acid at C-3′ went along with the further loss of the C-3 substituent (if substituted) or a water molecule. The loss of the fatty acyl moiety at C-3 was accompanied by the separate release of a water molecule (presumably from position C-4) and phosphoric acid (Figure 2c–e). Furthermore, cross-ring fragments, such as ^0,4^A_2_ and ^0,2^A_2_ ions (at *m*/*z* 1197 and 1257; Figure 2a,b) were detected for the 3-deacyl species, while only an ^0,4^A_2_ ion appeared (at *m*/*z* 1197 in Figure 2c and 1169 in Figure 2d,e) for the 3-acyl species.

In the lower mass region, next to the intact B_1_ ion (at *m*/*z* 1137 in Figure 2a–c and 1109 in Figure 2d,e), fragments formed by the serial release of the C-2′ secondary (giving the base peak at *m*/*z* 909), C-3′ secondary (*m*/*z* 680), and C-3′ primary fatty acid (*m*/*z* 454), as well as the C-4′ phosphate group (*m*/*z* 356) from the B_1_ ion could be identified. Moreover, a diacylated B_1_ ion (deficient of the C-3′ acyloxyacyl chain and the C-4’ phosphate group) was detected at *m*/*z* 556.

It is noteworthy that in Figure 2e of the native *E. coli* lipid A sample two intact (sodiated) B_1_ ions could be detected with similar intensities (i.e., at *m*/*z* 1029 and 1109), reflecting the simultaneous presence of two phosphorylation isomers, similarly as observed for the protonated molecule (Figure 1e). However, no further loss from the B_1_ ion at *m*/*z* 1029 originating from the 1-monophosphoryl isomer was observed (any loss from this ion was only detected by MS^3^ measurements).

Next, the intact [B_1_+ Na]^+^ ions present in the samples of the PHAD-504 standard and *E. coli* O83 bacterium were subjected to MS^3^ measurements (Figure S5). As expected, the fragmentation patterns closely resembled for the synthetic and bacterial lipid A samples with C-4′ phosphorylation (Figure S5a,b). However, the fragmentation pattern of the 1-monophosphoryl lipid A from *E. coli* (Figure S5c) showed differences, as the relative intensities of the fragments formed by secondary fatty acyl loss from the C-2′ and C-3′ positions were reversed, and (obviously) no ion due to the loss of a phosphate group appeared.

Overall, we conclude that the CID pattern of monosodiated lipid A contains common ion types with those seen in the CID pattern of both protonated and deprotonated lipid A. Figure S5 shows the principal similarities and differences between the fragmentation patterns of [M + Na]^+^ and [M + H]^+^ of 3D-PHAD. As a similarity, a B_1_ ion series is observed in both MS/MS mass spectra. However, in the case of the protonated molecule (Figure S6b), the base peak is the intact B_1_ ion, while for the sodium adduct (Figure S6a) it is that formed by a fatty acid loss (at the C-2′ branched position) from the intact B_1_ ion. The Y-type ion appears only for the protonated molecule. Another difference can be observed in the relative intensity of precursor ions, which is higher for the [M + Na]^+^ than for the [M + H]^+^ (at the same collision energy, 40 eV), indicating that the sodiated adduct is more stable than the protonated one. Furthermore, the loss of phosphoric acid and fatty acyl chains from the precursor, as well as the formation of cross-ring fragments can only be observed for the single sodium adduct ion and not for the protonated precursor. Meanwhile, it is precisely the serial fatty acid losses from the precursor and the A-type ion formation that make the CID mass spectrum of sodiated lipid A partly similar to that of the negative-ion MS/MS response of deprotonated lipid A.^12^

### CID Fragmentation Pattern of the Disodium Adduct of Monophosphorylated Lipid A

**Figure 3:**
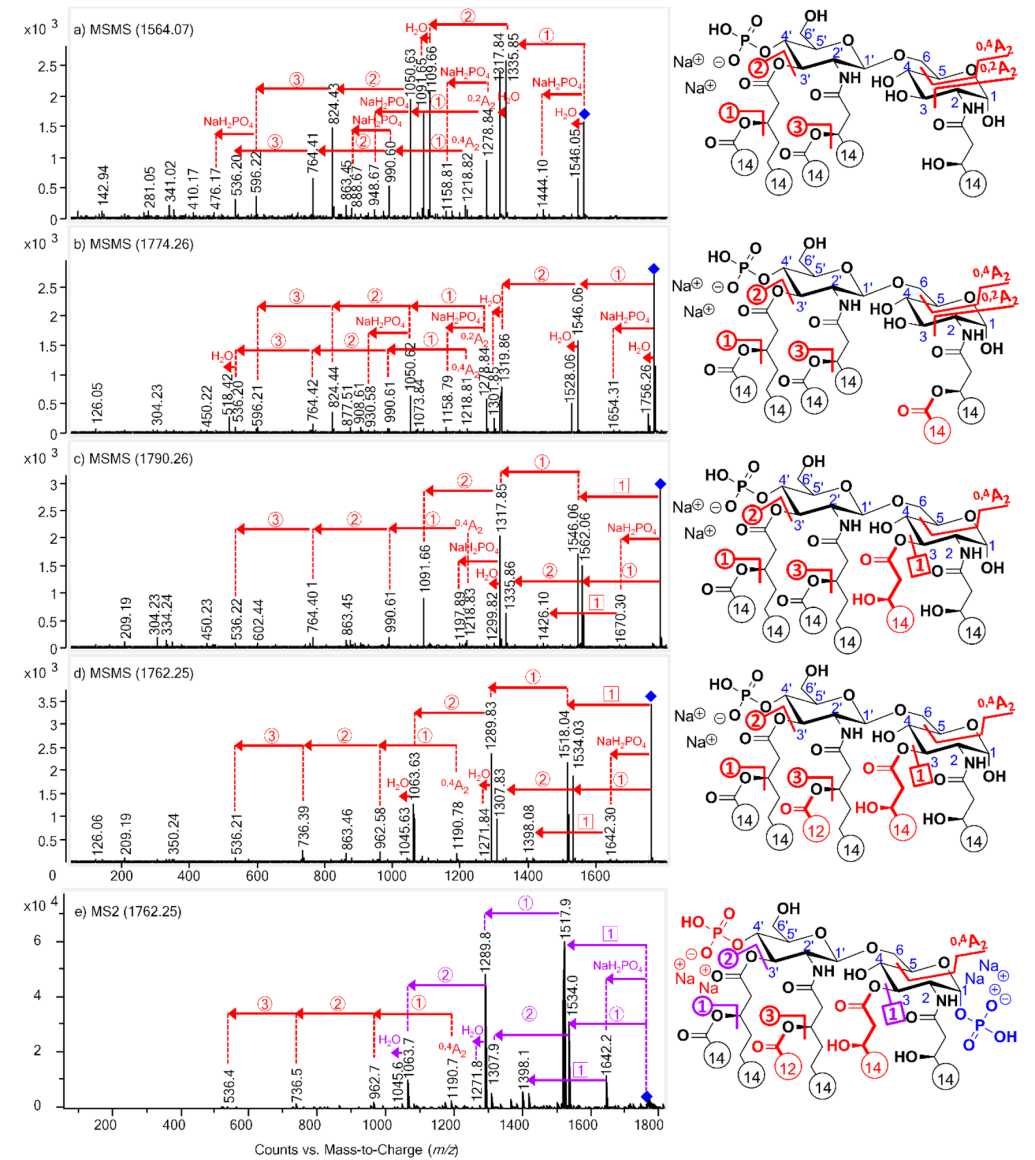
ESI-Q-TOF MS/MS mass spectra of [M – H + 2Na]^+^ lipid A precursor ions of (a) 3D-PHAD (*m*/*z* 1564), (b) 3D(6-acyl)-PHAD (*m*/*z* 1774), (c) PHAD (*m*/*z* 1790), (d) PHAD-504 (*m*/*z* 1762), and ESI ion trap MS/MS mass spectrum of (e) *E. coli* O83 (*m*/*z* 1762), with the indication of cleavage sites by numbers in circles or squares in the structures. Since both 1- and 4′-monophosphoryl species were present in the *E. coli* sample, its structure has been sketched carrying two phosphate groups for information purposes only. Red signs match with 4′-monophosphoryl species, blue signs match with 1-monophosphoryl species, and purple signs refer to both isomeric species. Fatty acyl chain lengths are given by numbers, and colored structural parts indicate the differences between the lipid A congeners.

Fragmentation pathways of the [M – H + 2Na]^+^ precursor ions of the standards and the native lipid A sample were also investigated by performing MS/MS analyses. A highly complex MS/MS fragmentation pattern was obtained for lipid A as disodium adducts (Figure 3), possessing several differences from that seen for the protonated molecule (Figure 1) and the monosodiated lipid A (Figure 2). For instance, inter-ring cleavage products (B- or Y-type ions) were not observed at all. Furthermore, not only intact ^0,2^A_2_ and ^0,4^A_2_ cross-ring fragments appeared (as seen for the monosodium adducts) but also those formed by sequential losses of the C-3′ secondary, C-3′ primary, and C-2′ secondary acyl residues. Note here as well that ^0,2^A_2_ ions were only detected for the 3-deacyl compounds (Figure 3a,b), while ^0,4^A_2_ ions appeared for all samples, except for the 1-monophosphoryl species within the *E. coli* sample. In fact, since no phosphate-free ^0,4^A_2_ ions were detected in Figure 3c–e, it could be assumed that the ^0,4^A_2_ ion series (as well as any A-type fragments) originated only from lipid A compounds carrying a C-4′ phosphate group.

For each disodium adduct, an abundant ion was produced in the MS/MS mass spectrum (i.e., at *m*/*z* 1336 for 3D-PHAD, 1546 for 3D-(6-acyl)-PHAD, 1562 for PHAD, and 1534 for PHAD-504 and *E. coli* in Figure 3a–e, respectively) resulting from the loss of the C-3′ secondary fatty acid (C14:0, 228 u) from the precursor. From that fragment, further loss of the C14:1 fatty acyl residue at C-3′ (226 u) generated a clearly detectable ion for all compounds, whereas water loss (18 u) from both of these fragments—and more importantly, from the precursor ion—could be observed only for the 3-deacyl standards (Figure 3a,b). For the 3-acyl lipid A, loss of the C-3 primary substituent (C14:0(3-OH), 244 u) from the precursor ion gave rise to another high-intensity ion (i.e., at *m*/*z* 1546 in Figure 3c and 1518 in Figure 3d,e, respectively), from which further loss of the C-3′ secondary fatty acid gave an abundant ion, as well (at *m*/*z* 1318 and 1290, respectively). This was followed by the loss of a C14:1 fatty acyl residue at C-3′ producing a relatively high-intensity ion (at *m*/*z* 1092 in Figure 3c and at *m*/*z* 1064 in Figure 3d,e). Furthermore, several ions due to water loss (18 u) and eliminations of the monosodium phosphate (NaH_2_PO_4_, 120 u) from the precursor or other product ions contributed the complexity of the fragmentation pattern of disodium adducts.

The most apparent observation from the study of the fragmentation pattern of the disodium adduct was that it highly resembles that of the deprotonated molecular ion of monophosphorylated lipid A.^12^ The main similarities and differences between the two CID mass spectra are demonstrated in Figure S7 by comparing the results of [M – H]^−^ and [M – H + 2Na]^+^ ions of 3D-PHAD. From both precursors, the same type of cross-ring cleavages and serial fatty acid losses arise, but fewer ions appear from the A-type fragment ion series in the case of the deprotonated lipid A. However, it should be noted that the CID fragmentation of a 4′-monophosphorylated lipid A also containing the C-3 fatty acyl substituent will not result in the formation of ^0,2^A_2_ ions in the case of the disodiated precursor, only in the case of the deprotonated one. Furthermore, the characteristic loss of the C-3′-linked fatty acyl chain as an acid and a ketene can only be seen during the dissociation of [M – H]^−^, whereas eliminations of the phosphate group (as NaH_2_PO_4_), and the loss of a water molecule from the precursor ion are only observed during the fragmentation of [M – H + 2Na]^+^.

### Utility of CID Fragmentation Regularities of Positively Charged Precursor Ions for the Structure Analysis of Monophosphorylated Lipid A

The combined analysis of at least two out of the three types of the positively charged precursors discussed above is of great benefit for the proper structure elucidation of different lipid A samples by positive-ion mode ESI-MS/MS. Altogether, the fragmentation regularities that aid in the identification of monophosphorylated lipid A molecules can be summarized as follows.

(1) The B_2_ ion—generated by the loss of either a water molecule or a phosphate group at C-1 of the [M + H]^+^ precursor ion—can be a mark used to distinguish between C-1 and C-4′ phosphorylation of lipid A. Accordingly, a B_2_ ion formed by phosphoric acid elimination (98 u) indicates C-1 phosphorylation, whereas a B_2_ ion arising from a water loss (18 u) indicates C-4′ phosphorylation. The appearance of both of these B_2_ ions in the MS/MS mass spectrum of [M + H]^+^ indicates that the sample is a mixture of C-1 and C-4′ phosphorylated lipid A. Furthermore, phosphoisomerism can be verified by observing two B_1_ ions—generated by the cleavage between the two glucosamine units—(with 80 u apart) instead of one in the MS/MS mass spectrum of either the [M + H]^+^ or the [M + Na]^+^ precursor ion (note that for [M + H]^+^ the B_1_ ion is the base peak in the MS/MS mass spectrum produced at lower collision energies, such as CE ≤ 40 eV in our experiments).

(2) The type of secondary fatty acid at the C-2′ position (if present) can be easiest identified by the mass difference between the B_1_ ion and a fragment next to it, formed by the loss of that fatty acid from the B_1_ ion (note that this secondary fatty acyl loss generates the base peak during the CID of the [M + Na]^+^ precursor, or by applying higher collision energies, such as CE ≥ 50 eV in our experiments, during the CID of the [M + H]^+^ precursor).

(3) Bond cleavage at C-3 occurs from both [M + Na]^+^ and [M – H + 2Na]^+^ precursors. Thus, detection of an abundant ion formed by a water loss (18 u) at C-3 from these sodium adducts indicates the absence of the C-3 primary acyl chain in lipid A, whereas the lack of this fragment in their MS/MS mass spectra defines a lipid A compound containing the C-3 acyl chain. In this latter case, the loss of the C-3 primary fatty acid will generate an abundant ion in the MS/MS mass spectra of both precursor ions.

(4) In the case of the [M – H + 2Na]^+^ precursor ion, the loss of the C-3 primary fatty acid is accompanied by the sequential loss of the C-3′ secondary and primary fatty acids, producing two other abundant ions in the MS/MS mass spectrum.

(5) Moreover, abundant ions are derived from the sequential loss of the C-3′ secondary and primary substituents from the [M – H + 2Na]^+^ precursor (note that in the case of a 3-deacyl compound, each of these ions are followed by a water loss).

(6) The assignment of the C-3′ secondary, C-3′ primary, and C-2′ secondary fatty acids can be confirmed by the presence of intact and nonintact cross-ring cleavage fragments, such as ^0,2^A_2_ (in the case of 3-deacyl) and ^0,4^A_2_ (in the case of both 3-deacyl and 3-acyl lipid A) in the MS/MS mass spectrum of the [M – H + 2Na]^+^ precursor. However, it is important to note that A-type cross-ring fragments are only observed for the C-4′ phosphorylated, and not for the C-1 phosphorylated lipid A, similarly as already demonstrated in the MS/MS analysis of deprotonated [M – H]^−^ precursor ions of lipid A phosphorylation isomers.^18^ Therefore, their presence or absence in the MS/MS mass spectrum of the [M + Na]^+^ and the [M – H + 2Na]^+^ precursors is indicative of the phosphorylation site, as well.

(7) The C-2 substitution on the reducing end of lipid A can be assigned from the Y_1_-type ion in the MS/MS mass spectrum of the [M + H]^+^ precursor ion. The *m*/*z* value of the Y_1_*, internal fragment ion (formed by the cleavage of the two glucosamine units and the glycosidic bond at C-1) can be obtained by calculating the mass difference between the precursor and the B_1_ ion, then adding [18 + 1 u] (for a C-4′ phosphorylated species) or [98 + 1 u] (for a C-1 phosphorylated species).

(8) In case the C-2 primary fatty acid is further substituted (forming an acyloxyacyl group), also the composition of the C-2 secondary fatty acid can be determined by the same mass difference between (i) the Y_1_* ion and a low-intensity ion next to the Y_1_* ion, and (ii) the B_2_ ion and a low-intensity ion next to the B_2_ ion.

## Conclusion

Most studies use negative-ionization mode MS/MS for the structural analysis of lipid A carrying an acidic phosphate group; however, those approaches prove unable to discern phosphorylation isomers and identify chimera mass spectra, which may for example arise during a shotgun mass spectrometry analysis or from coelution of phosphoisomers during an LC–MS separation. In this paper we have shown that positive-ion mode tandem mass spectrometry with low-energy CID allows for the full structural characterization of 4′-monophosphorylated compounds in natural mixtures of bacterial lipid A. The combined analysis of at least two out of the following three precursor ions, [M + H]^+^, [M + Na]^+^, and [M – H + 2Na]^+^, results in complementary structural information since it expands the diversity of possible cleavage sites induced by the conventional CID technique. Namely, information about the acyl linkages can be obtained at the C-2 primary and C-2/C-2′ secondary positions from the fragmentation pattern of the protonated lipid A and at the C-3′ secondary and C-3/C-3′ primary positions from the fragmentation pattern of the sodium-cationized forms of lipid A. After the assignment of the above-mentioned fatty acyl chains, the C-2′ primary linkage can be indirectly inferred. In addition, information on the phosphorylation site(s) is highly facilitated by the fragmentation pattern of the protonated or the monosodiated lipid A, permitting the assignment of the position of the phosphate group (i.e., C-1 or C-4′) and revealing the coexistance of phosphorylation isomers in complex mixtures. Particularly, the B_2_ ion formed from the [M + H]^+^ precursor is of great importance, as it directly points out the position of the phosphate group in lipid A (note that such a distinctive ion is absent in the negative-ion mode MS/MS mass spectrum of deprotonated lipid A). Knowledge on the exact location of phosphate group(s) and fatty acyl composition of the lipid A moiety is crucial for the proper recognition of immunological properties related to subtle structural modifications of this endotoxic molecule. Although the cleavage rules related to the 1-monophosphorylated lipid A species could not be fully described here (due to the lack of synthetic C-1 phosphoryl lipid A standards), this could be addressed in future studies by a previously reported nonaqueous capillary electrophoresis separation method^19^ to enable the resolution of coeluting phosphoisomers in native lipid A mixtures. Altogether, this study on positive-ion fragmentation regularities suggests the described method may be useful for qualitative analysis of native, heterogeneous lipid A samples using a single (positive) ESI ionization mode in an LC– or CE–MS/MS workflow.

## Supporting Information Available

Additional ESI-Q-TOF MS mass spectra, ESI ion trap MS^3^ mass spectra, comparison of ESI-Q-TOF MS/MS mass spectra of the monosodium adduct and the protonated molecule, and comparison of ESI-Q-TOF MS/MS mass spectra of the singly charged disodium adduct and the deprotonated molecule (PDF)
