# Supplementary material for: Complete Structural Elucidation of Monophosphorylated Lipid A by CID Fragmentation of Protonated Molecule and Singly Charged Sodiated Adducts
Source: J Am Soc Mass Spectrom. 2022 Dec 20;34(1):92–100. doi: 10.1021/jasms.2c00269 (PMC9817073; doi:10.1021/jasms.2c00269)
Supplement: Supplementary file 1 — js2c00269_si_001.pdf [file js2c00269_si_001.pdf]

Complete structural elucidation of monophosphorylated lipid A by CID fragmentation of protonated molecule and singly-charged sodiated adducts

Ibrahim Aissa[a], Ágnes Dörnyei[a], Viktor Sándor[b], Anikó Kilár[b]

[a] Department of Analytical and Environmental Chemistry, Faculty of Sciences, University of Pécs, Ifjúság útja 6, H-7624 Pécs, Hungary.

[b] Institute of Bioanalysis, Medical School and Szentágothai Research Centre, University of Pécs, Szigeti út 12, H-7624 Pécs, Hungary

Contents

Figure S1. ESI-Q-TOF MS mass spectra of a) 3D-PHAD b) 3D(6-acyl)-PHAD, c) PHAD, d) PHAD-504 and e) lipid A extracted from *E. coli* O83. Approximately 0.1 mg of each standard was dissolved in 1 mL of methanol/dichloromethane (70/30, v/v) mixture containing 5 mg of ammonium formate. Next, 300 µL of each sample was introduced into sealed glass vials and 700 µL of methanol was added. Additional 0.5-mg of NaCl was needed to promote the adduct formation for the bacterial extract. 2

Figure S2. Comparison of the ESI-Q-TOF MS mass spectra of the 3D-PHAD obtained in a) positive-ion and b) negative-ion modes. Approximately 0.1 mg of the sample was dissolved in 1 ml of methanol/dichloromethane (90/10, v/v) mixture. 3

Figure S3. ESI-ion trap MS^3 mass spectra of the intact $[B_1 + H]^+$ ion selected as a precursor at *m/z* 1115 of a) 3D-PHAD, b) 3D(6-acyl)-PHAD, c) PHAD, and d) at *m/z* 1087 of PHAD-504 with the indication of cleavage sites in the structures. Fatty acyl chain lengths are given by numbers. 4

Figure S4. ESI-ion trap mass spectra of the $[B_1 + H]^+$ ion obtained at MS^3 stage of the selected ion at a) *m/z* 1087 for the 4'-monophosphoryl species and b) *m/z* 1007 for the 1-monophosphoryl species from *E.coli* O83 lipid A, with the indication of cleavage sites in the structures. Red signs match with 4'-monophosphoryl, and blue signs with 1-monophosphoryl species. Fatty acyl chain lengths are given by numbers. 5

Figure S5. ESI-ion trap mass spectra of the $[B_1 + Na]^+$ ion obtained at MS^3 stage of the selected ion at a) *m/z* 1109 for PHAD-504, b) *m/z* 1109 for the 4'-monophosphoryl and c) *m/z* 1029 for the 1-monophosphoryl species from *E.coli* O83 lipid A, with the indication of cleavage sites in the structures. Red signs match with 4'-monophosphoryl, and blue signs with 1-monophosphoryl species. Fatty acyl chain lengths are given by numbers. 6

Figure S6. Comparison of ESI-Q-TOF MS/MS mass spectra of a) the sodium adduct $[M + Na]^+$ (*m/z* 1542) and b) the protonated molecular ion $[M + H]^+$ (*m/z* 1564) of 3D-PHAD. The similarities between the two fragmentation patterns are indicated in red, and the differences are shown in gray. ... 7

Figure S7. Comparison of ESI-Q-TOF MS/MS mass spectra of 3D-PHAD a) as a disodium adduct $[M – H + 2Na]^+$ (*m/z* 1564) measured in positive-ion mode, and b) as a deprotonated molecule $[M – H]^–$ (*m/z* 1518) measured in negative-ion mode (note that in this measurement only the ESI polarity was changed, all other experimental conditions remained the same). The similarities between the two fragmentation patterns are indicated in red, and the differences are shown in gray. 8

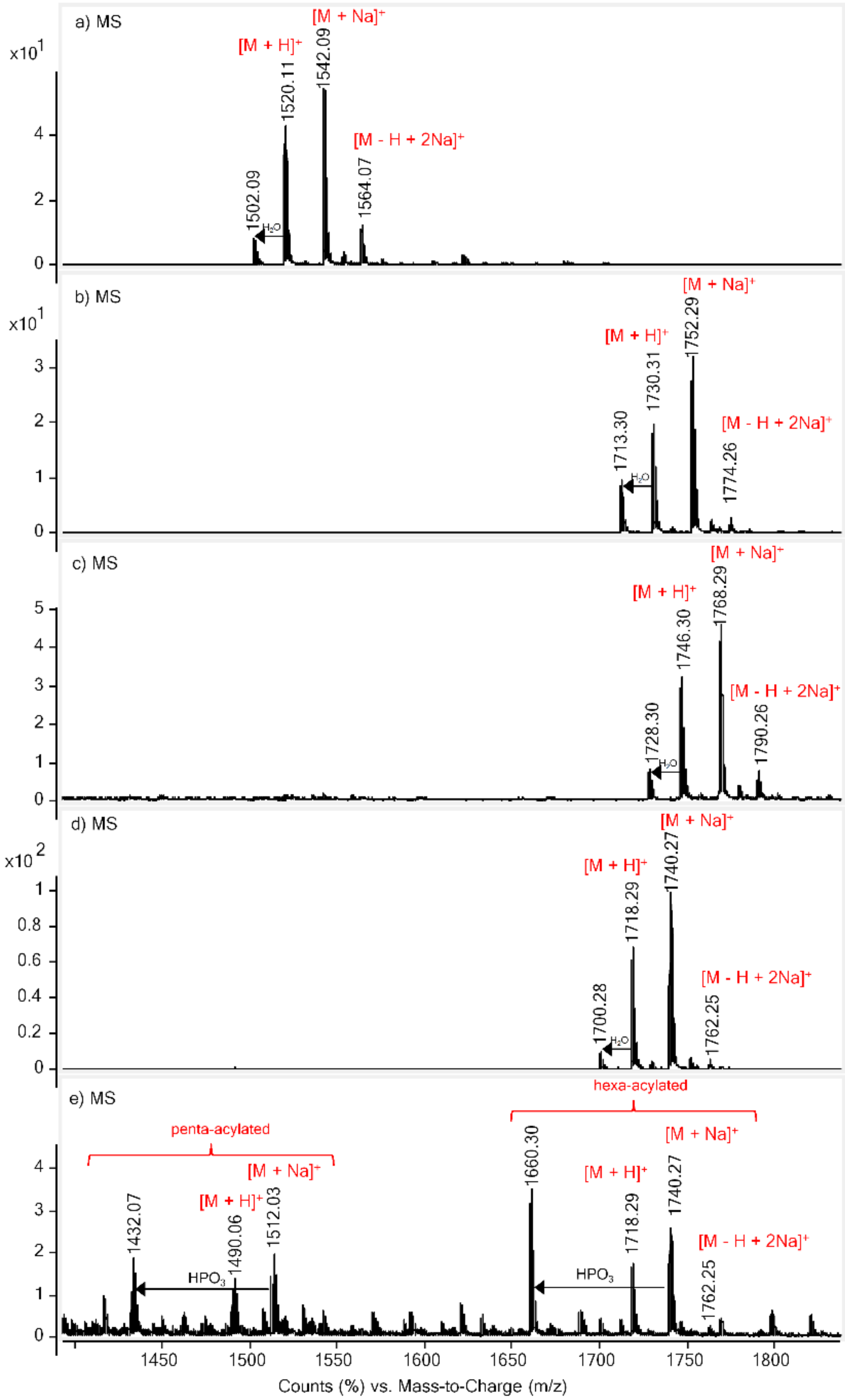

Figure S1. ESI-Q-TOF MS mass spectra of a) 3D-PHAD b) 3D(6-acyl)-PHAD, c) PHAD, d) PHAD-504 and e) lipid A extracted from *E. coli* O83. Approximately 0.1 mg of each standard was dissolved in 1 mL of methanol/dichloromethane (70/30, v/v) mixture containing 5 mg of ammonium formate. Next, 300 µL of each sample was introduced into sealed glass vials and 700 µL of methanol was added. Additional 0.5-mg of NaCl was needed to promote the adduct formation for the bacterial extract.

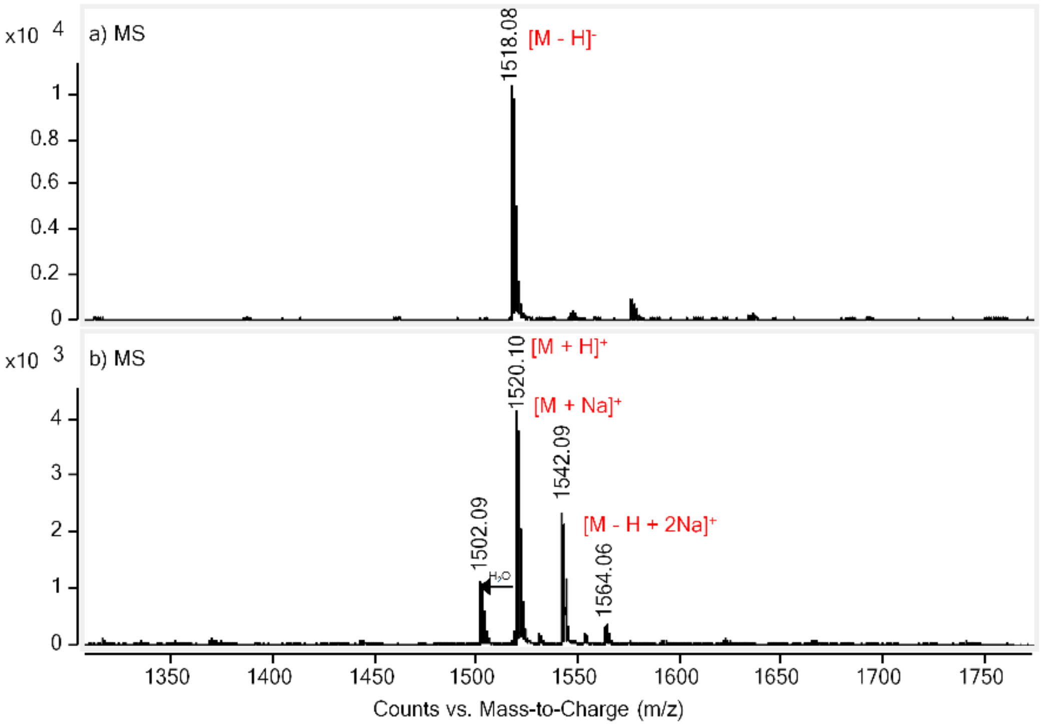

Figure S2. Comparison of the ESI-Q-TOF MS mass spectra of the 3D-PHAD obtained in a) positive-ion and b) negative-ion modes. Approximately 0.1 mg of the sample was dissolved in 1 ml of methanol/dichloromethane (90/10, v/v) mixture.

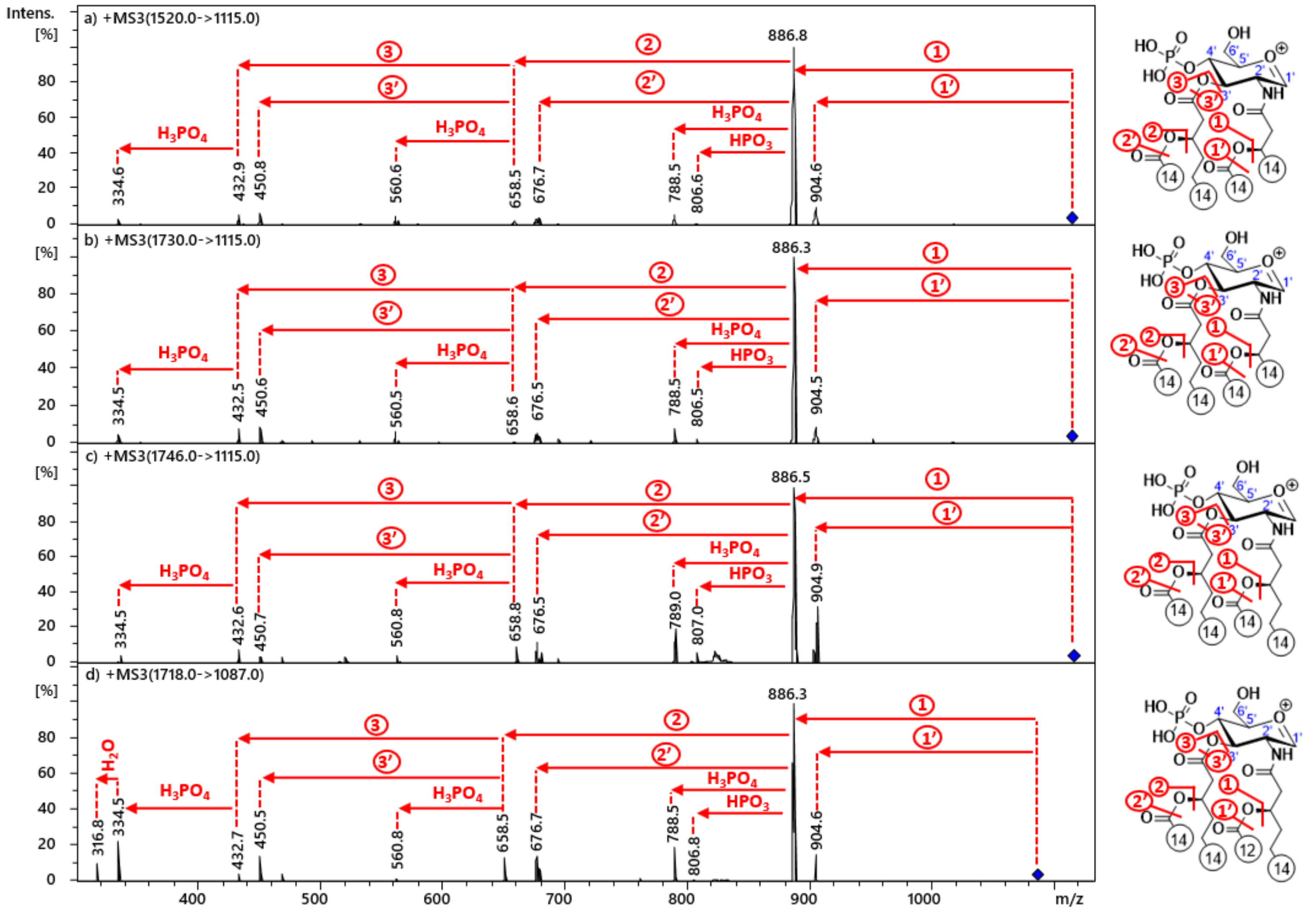

Figure S3. ESI-ion trap MS^3 mass spectra of the intact $[B_1 + H]^+$ ion selected as a precursor at *m/z* 1115 of a) 3D-PHAD, b) 3D(6-acyl)-PHAD, c) PHAD, and d) at *m/z* 1087 of PHAD-504 with the indication of cleavage sites in the structures. Fatty acyl chain lengths are given by numbers.

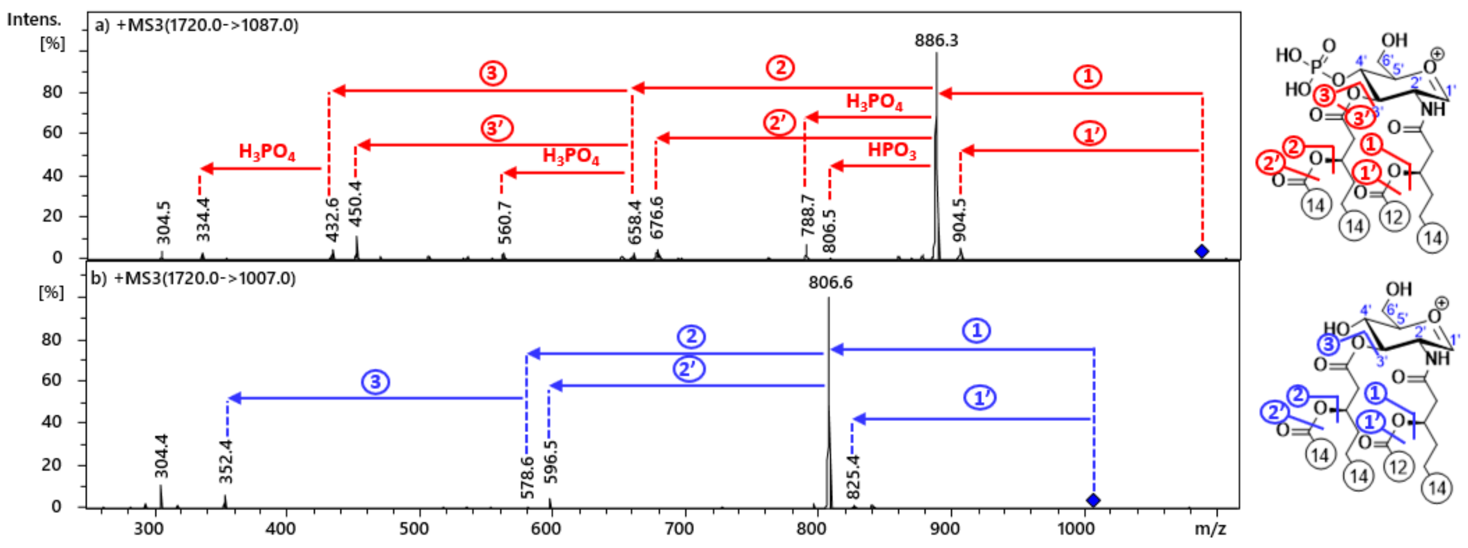

Figure S4. ESI-ion trap mass spectra of the $[B_1 + H]^+$ ion obtained at MS^3 stage of the selected ion at a) *m/z* 1087 for the 4'-monophosphoryl species and b) *m/z* 1007 for the 1-monophosphoryl species from *E.coli* O83 lipid A, with the indication of cleavage sites in the structures.
Red signs match with 4'-monophosphoryl, and blue signs with 1-monophosphoryl species. Fatty acyl chain lengths are given by numbers.

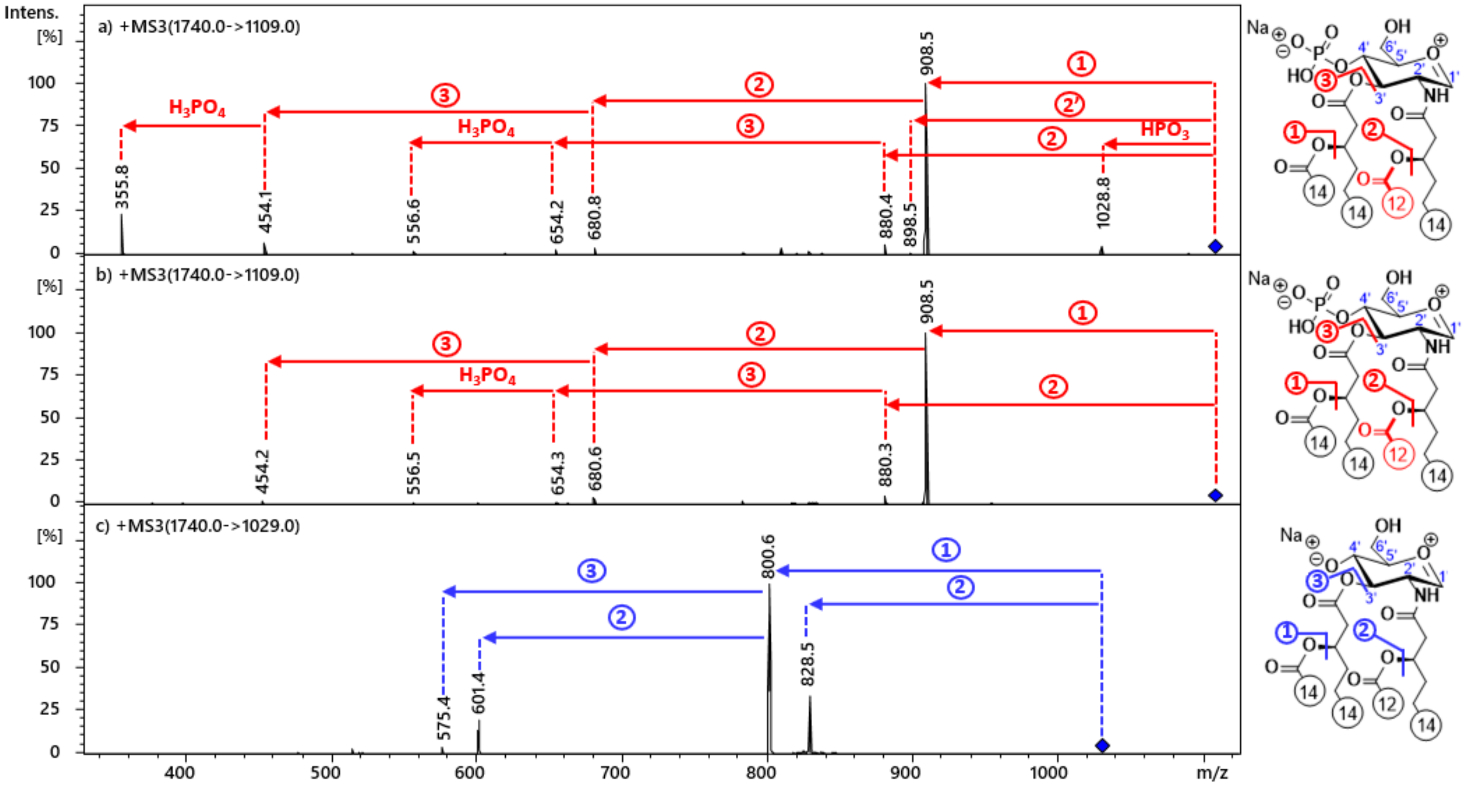

Figure S5. ESI-ion trap mass spectra of the $[B_1 + Na]^+$ ion obtained at MS^3 stage of the selected ion at a) *m/z* 1109 for PHAD-504, b) *m/z* 1109 for the 4'-monophosphoryl and c) *m/z* 1029 for the 1-monophosphoryl species from *E.coli* O83 lipid A, with the indication of cleavage sites in the structures. Red signs match with 4'-monophosphoryl, and blue signs with 1-monophosphoryl species. Fatty acyl chain lengths are given by numbers.

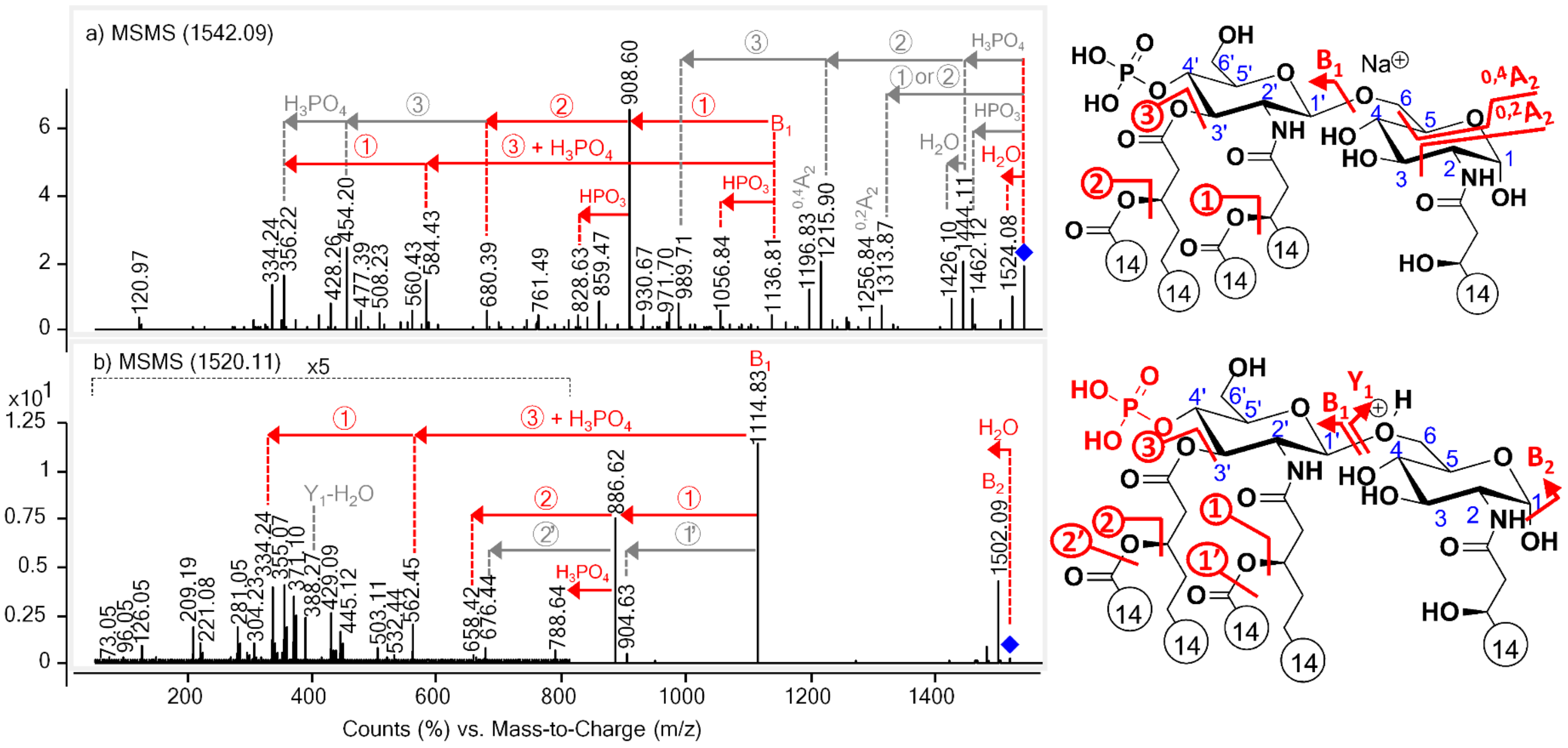

Figure S6. Comparison of ESI-Q-TOF MS/MS mass spectra of a) the sodium adduct $[M + Na]^+$ (*m/z* 1542) and b) the protonated molecular ion $[M + H]^+$ (*m/z* 1564) of 3D-PHAD. The similarities between the two fragmentation patterns are indicated in red, and the differences are shown in gray.

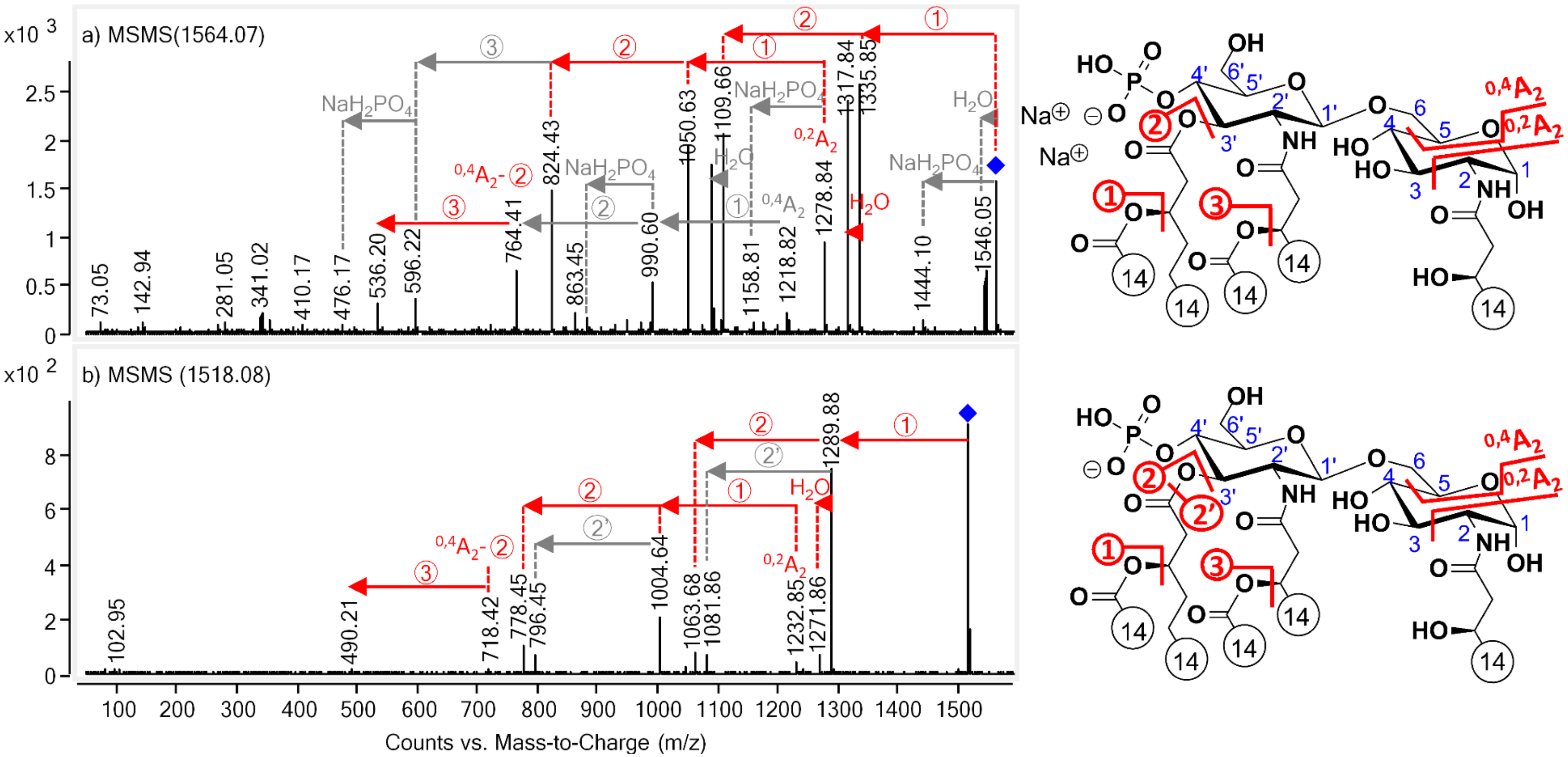

Figure S7. Comparison of ESI-Q-TOF MS/MS mass spectra of 3D-PHAD a) as a disodium adduct $[M - H + 2Na]^+$ (*m/z* 1564) measured in positive-ion mode, and b) as a deprotonated molecule $[M - H]^-$ (*m/z* 1518) measured in negative-ion mode (note that in this measurement only the ESI polarity was changed, all other experimental conditions remained the same). The similarities between the two fragmentation patterns are indicated in red, and the differences are shown in gray.
